# An Introduction to the Special Issue “Seeing Colors”

**DOI:** 10.1177/2041669518797392

**Published:** 2018-09-04

**Authors:** Mark W. Greenlee, John S. Werner, Christoph Wagner

**Affiliations:** University of Regensburg, Germany; University of California, Davis; University of Regensburg, Germany

Color vision is a prevalent sensory modality in modern society. We use color to communicate messages (“red means stop”), to highlight selected information, to denote the national identity (the colors in each country’s flag), and to enhance the salience of otherwise unnoticed information. It has a powerful role in grouping, which is why subway maps are often shown in color and virtually impossible to use when printed in gray scale. Color is abundant in nature and is used by animals to discriminate between ripe and unripe fruits or vegetables, edible versus nonedible foods, as well as between seasonal changes in foliage. We learn to associate certain colors with other sensory modalities, such as red with hot and blue with cold. Color also plays an important role in aesthetic appreciation. It is essential for pictorial works of art, architecture, design, cosmetics, and fashion. The scientific understanding of color vision goes back to the work of Sir Isaac Newton (1730) who made important observations about the nature of light and the realization that the proper understanding of color is in the constitution of the nervous system. In the 19th century, Hermann von Helmholtz suggested that different receptors in the eye were needed to differentiate between spectral colors. Working independently, Ewald Hering (1878) put forth the idea that color is encoded in an antagonistic fashion with the opponent axes green and red, yellow, and blue, as well as white and black. He proposed that these processes are antagonistic over space and time, in agreement with the earlier work of the French chemist Michel Eugene Chevreul (1839) who studied how the appearance of colored surfaces is altered by simultaneous viewing of another colored surface. Modern vision science has deepened our understanding of color vision.

The symposium on which this special issue is based brought together experts in color vision to discuss current theories of color and known phenomena related to color vision, including the underlying retinal and brain processes. These experts were invited to present their results in a manner that is understandable to an educated audience, who have little or no specialized knowledge about color vision. Our interdisciplinary approach united researchers from neuroscience, ophthalmology, vision and color science, cognitive psychology, art history, and philosophy.

This *i-Perception*’s special issue on “Seeing Colors” brought together 17 articles on various aspects of color vision. These range from the color appearance of negative afterimages (Anstis, 2017), to studies of color constancy (Morimoto, Mizokami, Yaguchi, & Buck, 2017; D. Weiss, Witzel, & Gegenfurtner, 2017), to methods of color appearance simulation (Kuriki, 2018), to the colors of The Dress (Feitosa-Santana, Lutze, Barrionuevo, & Cao, 2018), to the relationship between color memory and synesthesia (F. Weiss, Greenlee, & Volberg, 2018), as well as to the effect of color congruency on taste perception (Wieneke, Schmuck, Zacher, Greenlee, & Plank, 2018) and many more. We think this special issue puts together a wide range of approaches to the topic of Seeing Colors. We would like to acknowledge the financial support from the Hans Vielberth Foundation for the symposium that was held in September 2016 at the University of Regensburg.

## References

[bibr1-2041669518797392] Anstis S. (2017) Negative afterimages from flicker-augmented colors. i-Perception 8: 1–3, 2041669517699414 doi: 10.1177/2041669517699414. 10.1177/2041669517699414 PMC5426528 28529686

[bibr2-2041669518797392] Chevreul, M. E. (1839). *De la loi du contraste simultané des couleurs et de l'assortiment des objets colorés* [The principles of harmony and contrast of colours]. Paris, France: Chez Pitois-Levrault.

[bibr3-2041669518797392] Feitosa-Santana C. Lutze M. Barrionuevo P. A. Cao D. (2018) Assessment of #TheDress with traditional color vision tests: Perception differences are associated with blueness. i-Perception 9: 1–17, 2041669518764192 doi: 10.1177/2041669518764192. 10.1177/2041669518764192 PMC5937631 29755724

[bibr4-2041669518797392] Hering E. (1878) Zur Lehre von Lichtsinn *[On the Light Sense]*, Berlin, Germany: Akademie der Wissenschaften.

[bibr5-2041669518797392] Kuriki I. (2018) A novel method of color appearance simulation using achromatic point locus with lightness dependence. i-Perception 9: 1–16, 2041669518761731 doi: 10.1177/2041669518761731. 10.1177/2041669518761731 PMC5937632 29755723

[bibr6-2041669518797392] Morimoto T. Mizokami Y. Yaguchi H. Buck S. L. (2017) Color constancy in two-dimensional and three-dimensional scenes: Effects of viewing methods and surface texture. i-Perception 8: 1–20, 2041669517743522 doi: 10.1177/2041669517743522. 10.1177/2041669517743522 PMC5721973 29238513

[bibr7-2041669518797392] Newton I. (1730) Opticks: A treatise of the reflections, refractions, inflections and colours of light, 4th ed London, England: William Innys.

[bibr8-2041669518797392] Weiss D. Witzel C. Gegenfurtner K. (2017) Determinants of colour constancy and the blue bias. i-Perception 8: 1–29, 2041669517739635 doi: 10.1177/2041669517739635. 10.1177/2041669517739635 PMC5768282 29348910

[bibr9-2041669518797392] Weiss F. Greenlee M. W. Volberg G. (2018) Gray bananas and a red letter A — From synesthetic sensation to memory colors. i-Perception 9: 1–26, 2041669518777515 doi: 10.1177/2041669518777515. 10.1177/2041669518777515 PMC5985554 29899968

[bibr10-2041669518797392] Wieneke L. Schmuck P. Zacher J. Greenlee M. W. Plank T. (2018) Effects of congruent and incongruent stimulus colour on flavour discriminations. i-Perception 9: 1–11, 2041669518761463 doi: 10.1177/2041669518761463. 10.1177/2041669518761463 PMC5937635 29755721

